# Assessment of the Antibiotic Resistance Profile, Genetic Heterogeneity and Biofilm Production of Methicillin-Resistant *Staphylococcus aureus* (MRSA) Isolated from The Italian Swine Production Chain

**DOI:** 10.3390/foods9091141

**Published:** 2020-08-19

**Authors:** Pedro Rodríguez-López, Virginia Filipello, Pierluigi Aldo Di Ciccio, Alessandra Pitozzi, Sergio Ghidini, Federico Scali, Adriana Ianieri, Emanuela Zanardi, Marina Nadia Losio, Ancuta Cezara Simon, Giovanni Loris Alborali

**Affiliations:** 1 Department of Food and Drug, Università di Parma, Strada del Taglio 10, 43126 Parma, Italy; pedro.rodriguezlopez@unipr.it (P.R.-L.); sergio.ghidini@unipr.it (S.G.); adriana.ianieri@unipr.it (A.I.); emanuela.zanardi@unipr.it (E.Z.); 2 Istituto Zooprofilattico Sperimentale della Lombardia e dell’Emilia Romagna—Headquarters, Via A. Bianchi, 9, 25124 Brescia, Italy; virginia.filipello@izsler.it (V.F.); alessandra.pitozzi@izsler.it (A.P.); federico.scali@izsler.it (F.S.); marinanadia.losio@izsler.it (M.N.L.); giovanni.alborali@izsler.it (G.L.A.); 3 Dipartimento di Scienze Veterinarie, University of Turin, Largo P. Braccini 2, Grugliasco, 10095 Turin, Italy; pierluigialdo.diciccio@unito.it

**Keywords:** antibiotic susceptibility, antibiotic resistance, food safety, food hygiene, MRSA, prevalence, public health, *Staphylococcus aureus*, survey

## Abstract

The main aim of the present study was to evaluate the level of antibiotic resistance, prevalence and virulence features of methicillin-resistant *Staphylococcus aureus* (MRSA) isolated from heavy swine at abattoir level and farming environments in Lombardy (Northern Italy). With this scope, 88 different heavy swine farms were surveyed, obtaining a total of *n* = 440 animal swabs and *n* = 150 environmental swabs. A total of *n* = 87 MRSA isolates were obtained, with an overall MRSA incidence of 17.50% (*n* = 77) among animal samples and a 6.67% (*n* = 10) among environmental. Molecular characterisation using multilocus sequence typing (MLST) plus *spa*-typing showed that sequence type ST398/t899 and ST398/t011 were the most commonly isolated genotypes, although other relevant sequence types such as ST1 or ST97 were also found. A lack of susceptibility to penicillins, tetracycline and ceftiofur was detected in >91.95, 85.05 and 48.28% of the isolates, respectively. Resistance to doxycycline (32.18%), enrofloxacin (27.59%) and gentamicin (25.29%) was also observed. Additionally, a remarkable level of antibiotic multiresistance (AMR) was observed representing a 77.01% (*n* = 67) of the obtained isolates. Genetic analysis revealed that 97.70% and 77.01% of the isolates harboured at least one antibiotic resistance or enterotoxin gene, respectively, pointing out a high isolate virulence potential. Lastly, 55.17% (*n* = 48) were able to produce measurable amounts of biofilm after 24 h. In spite of the current programmes for antibiotic reduction in intensively farming, a still on-going high level of AMR and virulence potential in MRSA was demonstrated, making this pathogen a serious risk in swine production chain, highlighting once more the need to develop efficient, pathogen-specific control strategies.

## 1. Introduction

Currently, the emergence of antimicrobial-resistance (AR) in bacteria represents a major challenge for public health. Poor biosecurity practises and the use, misuse and overuse of antimicrobial agents as therapeutic drugs in human medicine, animal farming and in agricultural settings may apply an environmental pressure favouring the selection of AR bacteria [1]. In addition to this fact, the inappropriate use of biocides in farms and food industries may select for a subset of resistant population or even induce resistance to unrelated and clinically relevant antibiotics, in a phenomenon known as antimicrobial cross-resistance [2,3,4]. To date, the contribution of the contaminated environment to the spread of antimicrobial-resistant microorganisms is not fully understood. In this context, a “one health” approach with an integrated multi-sectorial effort involving animals and their surrounding environment is needed to tackle the spread of AR in the food chain. In detail, the presence of bacterial biofilms can also promote the emergence of AR by spontaneous mutation of bacteria or by means of plasmid-mediated horizontal gene transfer [5].

Methicillin-resistant *Staphylococcus aureus* (MRSA) is the most commonly identified antibiotic-resistant pathogen worldwide [6]. It is resistant to almost all β-lactams and can also show resistance to other major antimicrobial classes such as fluoroquinolones [3,7]. The livestock-associated MRSA (LA-MRSA) is considered a serious concern for the risks of zoonotic transmission, not only to people with occupational livestock exposure [8,9], but also to the community through the food chain [10]. The foremost common LA-MRSA worldwide is the sequence type (ST) 398 belonging to clonal complex (CC) 398 and the main reservoir for this clone are pigs [11,12]. After its first isolation from pigs in France [13], ST398 was increasingly detected throughout Europe. MRSA infection or colonisation in pigs may results in economic losses in the food industry and could represent a zoonotic risk for humans in contact with infected or colonised animals [14,15,16].

Even though MRSA transmission is mainly due to human-to-human contact, several studies have demonstrated that the animal–human transmission (i.e., at abattoir level) cannot be neglected due to its implications in MRSA spreading [17,18,19]. Italian swine farming is mainly aimed at the production of heavy swine for cured meat production, slaughtered at 165–170 Kg live weight i.e., approximately 9 months of life. The major pig production basin is located in three regions of Northern Italy i.e., Emilia Romagna, Lombardy and Piedmont accounting for 78.59% of Italian pigs according to 2019 data [20]. Monitoring of MRSA strains in Italian pig production chain is thus of upmost importance to obtain early indication data of local prevalence and to evaluate changes in AMR and to diminish its dissemination.

Some studies reported a high prevalence (34.9–38.1%) of LA-MRSA from pig holdings in Italy [21,22]. Furthermore, an estimated MRSA prevalence of 37.6% was recently reported in slaughtered pigs of two industrial abattoirs in Southern Italy [23]. The presence of biofilm in farm or food processing environment could play an important role in MRSA spreading throughout the food chain [24]. These structures are characterised by an enhanced capacity to endure harsh environmental conditions such as cleaning and disinfection protocols commonly used in food-related premises and therefore acting as a pathogen reservoir [25,26,27]. With this regard, the study carried out by Vergara et al. [28] demonstrated that MRSA strains isolated from the food sector can form biofilm on various food-related surfaces when they are exposed to conditions simulating those in food processing plants. Additionally, the biofilm-forming ability of MRSA that are potentially enterotoxin producers should be of concern for food safety [28]. Currently, research studies on the AMR and ability of biofilm formation of MRSA are more common in strains coming from clinical scenarios when compared to those involving strains isolated from food-related environments. Consequently, it is necessary to also monitor the prevalence and biofilm formation capacity of MRSA present in the food supply chain.

Considering the great concern of the presence and dispersion of LA-MRSA clones in food-producing animals such as pigs, which have a zoonotic potential, and their high isolation rate from Italian intensive pig farms, the aims of this survey were: (i) to determine the antimicrobial-resistance and heterogeneity of MRSA found in Italian heavy swine production chain, (ii) to analyse the population structure of these MRSA strains and (iii) to provide data on biofilm formation of the obtained isolates.

## 2. Materials and Methods

### 2.1. Sampling Design

A survey was carried out in a total of 88 heavy pig (*Sus scrofa domesticus*) farms (industrial livestock farming) located in Lombardy (Northern Italy) between 2016 and 2018. Overall, these farms represent about 20% of the total Italian heavy swine production and slaughtered animals (19.93% in 2018 and 19.75% according to latest Eurostat data [29]). From each farm, 5 different animals were randomly selected and animal samples were collected at abattoir level after stunning by rubbing a neck area of 50 × 50 cm using a sterile cotton swab, 1 per animal, thus obtaining a total number of *n* = 440 samples.

In addition to this, in 50 pig farms, different from those previously sampled, environmental samples were collected on a voluntary basis. Specifically, sterile cotton swabs were used to pick up samples from 3 different locations, i.e., barn, aeration device and feeder, obtaining a total number of samples of *n* = 150.

In all cases, swabs were then stored at 4 °C and processed within 6 h after sampling.

### 2.2. Isolation of Suspected MRSA

Samples were enriched in brain hearth infusion (BHI, Oxoid, Hampshire, England) containing 6.5% NaCl (Biolife, Milan, Italy) and incubated for 18–24 h at 37 °C. Next, 0.1 mL of the resulting broth was plated onto CHROMID^®^ MRSA plates (bioMérieux, Marcy-l’Étoile, France) and Baird-Parker agar supplemented with rabbit plasma fibrinogen (BP-RPF; Oxoid) following the ISO 6888-2 norm [30]. Suspected colonies were further subjected to Gram staining, coagulase and urease test. Finally, for each sample, one suspected colony was struck onto tryptone soy agar (TSA, Oxoid) and incubated at 37 °C for 24 h for further analysis.

### 2.3. MRSA Confirmation

Confirmation of suspected colonies was performed via detection of *nuc* and *me*cA genes using a duplex colony PCR following the protocol reported by Pichon et al. [31]. Amplicons were resolved in a 1.5% agarose gel (Agarose Multi-Purpose, Roche) stained with GelRed (Biotium, Hayward, CA, USA) using 1 X Tris-Acetate-EDTA (TAE) buffer (Merck KGaA, Darmstadt, Germany). GeneRuler 100 bp Plus DNA Ladder (ThermoFisher Scientific, Vilnius, Lithuania) was used as a molecular marker and finally visualisation was carried out under UV light.

In the case of positive PCR confirmation, working cultures were prepared by mixing a loopful from TSA plates into a 2 mL sterile cryogenic vial containing 1 mL of sterile 50% glycerol. Cultures were stored at −20 °C for further analysis.

### 2.4. Antibiotic Resistance Profiling of Confirmed MRSA Isolates

#### 2.4.1. Disk Diffusion Assays

Isolates identified as MRSA were tested for susceptibility to a panel of 14 antimicrobial agents using the disk diffusion method following the Clinical & Laboratory Standards Institute indications [32].

The antibiotic disks (Liofilchem) were as follows: amoxicillin/clavulanic acid (AMC; 30 µg), ampicillin (AMP; 10 µg), ceftiofur (FUR; 30 µg), cephalothin (KF; 30 µg), chloramphenicol (C; 30 µg), doxycycline (DXT; 30 µg), enrofloxacin (ENR; 5 µg), florfenicol (FFC; 30 µg), gentamicin (CN; 10 µg), oxacillin (OX; 1 µg), penicillin (P; 30 µg), trimethoprim/sulfamethoxazole (SxT; 25 µg), tetracycline (TET; 30 µg) and tiamulin (T; 30 µg). A maximum of 6 disks were used in each plate and were incubated at 37 °C for 24 h. After incubation, inhibition zones were measured with a calliper and isolates were classified as resistant or non-resistant. Of note, those classified as intermediate were included in the latter category. Isolates were considered as antibiotic-multiresistant (AMR) if presented resistance to three or more antibiotic categories [33].

#### 2.4.2. Minimum Inhibitory Concentration Assays

In those determined as AMR isolates, the minimum inhibitory concentrations (MIC) to those antibiotics considered as critically important for both veterinary and human medicine [34,35] were determined using the following MIC test strips (Liofilchem): AMC (0.016–256 mg/L), CN (0.064–1024 mg/L), DXT (0.016–256 mg/L), ENR (0.002–32 mg/L), FFC (0.016–245 mg/L), SxT (0.002–32 mg/L), TET (0.016–245 mg/L). Plates were incubated at 37 °C, 24 h and results were compared using available data at the European Committee on Antimicrobial Susceptibility Testing (EUCAST) [36].

#### 2.4.3. Antimicrobial Resistance Genes Determination

Five different in-house customised PCR protocols were used to determine the presence of genes involved in antimicrobial resistance using primers targeting for genes related to resistance to β-lactams (*bla*Z [17]), tetracyclines (*tet*L, *tet*M [17]), quinolones (*nor*A [37]), amphenicols (*cfr* [38], *fex*A [39]), aminoglycosides (*aac*A*-aph*D, *aad*D [17]), pleuromutilins (*vga*A [40] and *vga*E [41]), trimethoprim (*dfr*A, *dfr*D, *dfr*K [17]) and macrolides (*erm*T [17]). A complete list of primer names, sequences, expected product and references is provided in Table S1. In all cases, extraction of total DNA and amplicon resolving was performed as described in Section 2.3. PCR reactions were carried out in a final volume of 15 µL containing 1X Hot Star Master Mix (Qiagen, Hilden, Germany), 0.5 µM of each primer and 3 µL of template DNA. PCR conditions were as follows:

- Multiplex 1 (*aac*A-*aph*D, *tet*L, *tet*M, *dfr*A, *dfr*D): 95 °C (5 min), followed by 32 cycles of 95 °C (30 s), 45 °C (45 s), 72 °C (1 min) and a final extension at 72 °C (10 min).
- Multiplex 2 (*bla*Z, *dfr*K, *vga*E): 95 °C (5 min), followed by 32 cycles of 95 °C (30 s), 47 °C (45 s), 72 °C (50 s) and a final extension at 72 °C (10 min).
- Multiplex 3 (*erm*T, *nor*A, *vga*A): 95 °C (5 min), followed by 31 cycles of 95 °C (30 s), 49 °C (40 s), 72 °C (1 min) and a final extension at 72 °C (10 min).
- Multiplex 4 (*aad*D, *cfr*): 95 °C (5 min), followed by 31 cycles of 95 °C (30 s), 52 °C (40 s), 72 °C (50 s) and a final extension at 72 °C (10 min).
- PCR 5 (*fex*A): 95 °C (5 min), followed by 31 cycles of 95 °C (30 s), 56 °C (40 s), 72 °C (1 min) and a final extension at 72 °C (10 min).

### 2.5. Enterotoxin Gene Determination

The presence of genes related with staphylococcal enterotoxins A (*sea*), B (*seb*), C (*sec*), D (*sed*) and E (*see*) [42] was determined using the primer sets listed in Table S1. A multiplex PCR protocol was followed as described by Bianchi et al. [43]. In all cases, PCR reaction products were resolved as described in Section 2.3.

### 2.6. MRSA Molecular Subtyping and Phylogenetic Analysis

Samples identified as MRSA were subtyped with multilocus sequence typing (MLST) as described by Enright et al. [44]. To determine the sequence types (STs), data available on the *Staphylococcus aureus* MLST database [45] were used. For the *spa* typing, the *spa* gene of MRSA strains was amplified by PCR as described by Shopsin et al. [46], and *spa* subtypes were determined with Ridom StaphType software (Ridom GmbH Würzburg, Münster, Germany). A 3500xL genetic analyser (Applied Biosystems, Foster City, CA, USA) was used to obtain all the DNA sequences. Following this, phylogenetic analysis was conducted following the protocol described previously by Filipello et al. [19].

### 2.7. Quantification of Biofilm Production

The ability of MRSA isolates to produce biofilms on flat-bottomed polystyrene microplates was determined according to the protocol described by Stepanović et al. [47] with slight modifications. In all cases, all experiments were repeated in triplicate.

First, reactivation of the cells was carried out by transferring 100 µL of working cultures into 5 mL of TSB and incubated overnight at 37 °C. Next, cultures were adjusted to Abs_700_ = 0.1 ± 0.001 in sterile saline using UV visible spectrophotometer (Varian SII Scan Cary 100 Spectrophotometer—Agilent Technologies, Santa Clara, CA, USA), corresponding to a concentration about 10^8^ CFU/mL, according to previous calibrations. Adjusted cultures were 1:100 diluted in sterile fresh TSB + 1% glucose (Merck, Darmsatdt, Germany) reaching a final concentration of about 10^6^ CFU/mL. In all cases, inocula cellular density was checked by plating onto TSA.

Following this, 200 µL of inoculum was transferred to seven wells of a sterile 96-well flat-bottomed polystyrene plate (ThermoFisher Scientific). In all cases 200 µL of sterile TSB + 1% glucose was used as negative control. Then plates were covered and incubated for 24 h at 37 °C in static conditions. After incubation, bulk culture was pipetted out, and each well was carefully washed three times with 300 µL of sterile phosphate-buffered saline (PBS, Oxoid) in order to remove loosely attached cells. Samples were then fixed at 60 °C for 1 h and stained with 150 µL of 2% crystal violet solution (CV; Condalab, Madrid, Spain) for 15 min. Excess staining was removed by washing wells with running tap water and allowed to air dry. Finally, 150 µL of 95% ethanol was added to each well to de-stain the wells and let to dwell for 30 min at room temperature.

Quantitative analysis was performed by measuring the absorbance at 540 nm using a microplate reader (Microscan FC, Thermo Scientific, Boston, MA, USA). All results were expressed by calculating the biofilm production index (BPI) as follows: OD (OD mean sample–OD negative control), whereas OD for the negative control was measured as OD_nc_: OD negative control + (3 × standard deviation of the negative control). Strains showing ability to produce biofilm were classified as weak (OD_nc_ < OD ≤ 2 × OD_nc_), moderate (2 × OD_n c_ < OD ≤ 4 × OD_nc_) or strong (OD > 4 × OD_nc_) biofilm producers [47].

### 2.8. Statistical Analysis

A two-sided chi-square (Χ^2^) test using IBM SPSS Statistics for Windows, Version 23.0 (IBM Corp. Armonk, NY, USA) was conducted to determine the correlation between the biofilm formation ability and the antibiotic multiresistance of the MRSA isolates. A *p*-value of <0.05 was deemed to be significant.

## 3. Results

### 3.1. MRSA Detection

A total of 440 animal samples (i.e., swabs) obtained from 88 different fattening units at abattoir level were screened in order to determine the presence of MRSA by means of enrichment culture and isolation using agar plating with PCR confirmation of *mec*A gene. Overall, 42 (47.72%) resulted positive for MRSA. In samples from animals after stunning, positivity was detected in 17.50% (*n* = 77) of the analysed samples

Regarding environmental samples (*n* = 150) from 50 different fattening units, positivity was found in 14% (*n* = 7) of the units surveyed. Molecular confirmation via *mec*A detection gave MRSA positivity in 6.67% (*n* = 10) of the environmental samples collected. Consequently, the overall incidence of MRSA-positive samples was 14.75% (*n* = 87).

### 3.2. Antibiotic Resistance Profiles of MRSA Isolates

#### 3.2.1. Phenotypic Resistance

**Figure 1:**
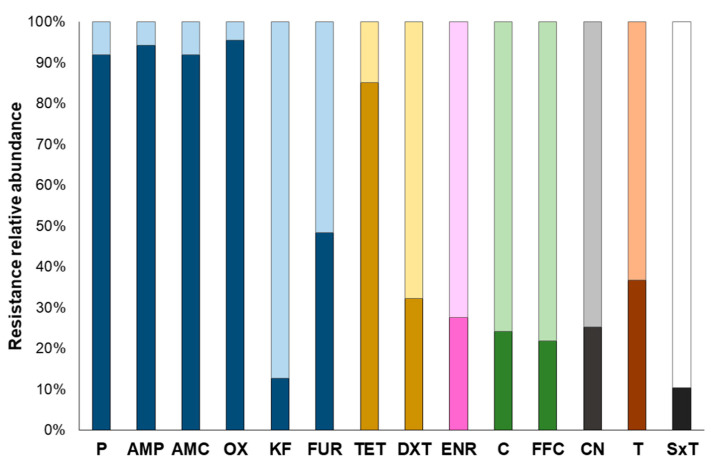
Relative abundances of observed antibiotic resistances in MRSA isolates (*n* = 87) determined using the disk diffusion (Kirby–Bauer) assay. In each bar depicting each one of the antibiotics used, the darker parts represent the percentage of resistant isolates, whereas the lighter parts are indicative of those classified as non-resistant. Antibiotic classes are represented with different colour bars as follows: blue: β-lactams (P: penicillin; AMP: ampicillin; AMC: amoxicillin/clavulanic acid; OX: oxacillin; KF: cephalothin; FUR: ceftiofur); yellow: tetracyclines (TET: tetracycline; DXT: doxycycline); pink: fluoroquinolones (ENR: enrofloxacin); green: amphenicols (C: chloramphenicol; FFC: florfenicol), grey: aminoglycosides (CN: gentamycin); brown: pleuromutilins (T: Tiamulin); black and white: folate inhibitors (SxT: sulfamethoxazole/trimethoprim).

High resistance in β-lactams, especially penicillins (>91% of the isolates), 3rd generation cephalosporin (i.e., ceftiofur, 48.28%), and tetracycline (85.05%), was observed in MRSA isolates (Figure 1). With regard to the rest of the antibiotics tested, percentage of resistance among MRSA isolates ranged between 36.78% (*n* = 32) in tiamulin (pleuromutilin) and 10.34% (*n* = 9) in the case of trimethoprim/sulfamethoxazole (Figure 1). Of note, almost all MRSA isolates (85 out of 87) tested displayed resistance to at least three different antibiotics. The only two exceptions were isolate SA14 exhibiting only resistance to oxacillin and isolate SA4 with no phenotypic antibiotic resistance detected (Table S2).

Antibiotic multiresistance (AMR), i.e., resistance to three or more classes of antibiotics [33], was observed in 77.01% (*n* = 67) of the MRSA isolates obtained. Taking a closer look, some of the antibiotic patterns were repeated among the isolates. Specifically, a 62.69% (*n* = 42) of the isolates presented an antibiotic resistance profile that was present in at least two of the isolates (Table 1). It is important to highlight that these observed patterns were not exclusively associated to a given genotype (i.e., ST/*spa*-type) but a genetic heterogeneity was observed (Table 1). Of note, among those that belonged to the same genotype, differences either in the antimicrobial genes, in the enterotoxin genes or both were detected, indicating that even though closely related, the isolates were different to one another (results not shown).

Maximum level of AMR, i.e., lack of susceptibility upon 12 of the 15 molecules tested, representing six different classes, was displayed by isolates SA3 (P-AMC-OX-AMP-FUR-TE-DXT-C-FFC-CN-T-SxT) and SA15 (P-AMC-OX-AMP-FUR-TE-DXT-ENR-C-FFC-CN-T). Of note, both were isolated from environmental sources (Table 2, Table S2).

#### 3.2.2. Minimum Inhibitory Concentration (MIC) Assays

Determination of MIC values in AMR isolates to antibiotics, considered as highly important or critical for antimicrobial chemotherapy in human (CN), veterinary (DXT, TET) or both (AMC, ENR, SxT, FFC), was determined in multiresistant isolates by means of miniaturised systems (MIC strips). Results showed the highest MIC values in TET (MIC_TET_ ≥ 6 mg/L), followed by DXT (MIC_DXT_ ≥ 8 mg/L), SxT (MIC_SxT_ ≥ 4 mg/L) and CN (MIC_CN_ > 1.5 mg/L), which are all above the available EUCAST resistance breakpoint values [36]. The range of MIC values for those antibiotics in which EUCAST does not provide a specific breakpoint value were as follows: MIC_AMC_ = 0.19–24 mg/L; MIC_ENR_ = 0.2–>32 mg/L; MIC_FFC_ = 3–>256 mg/L).

#### 3.2.3. Presence of Antibiotic Resistance Genes

Results obtained demonstrated that all MRSA, except isolate SA6, turned out to be positive in PCR assays and carried at least one antibiotic resistance gene (Table 2). Specifically, 82.75% (*n* = 72) of the MRSA isolates carried *dfr*A, followed by *tet*M (81.61%, *n* = 71), *bla*Z (78.16%, *n* = 68) and *nor*A (77.01%, *n* = 67). In a lesser extent, *dfr*D, *erm*T and *cfr* genes were detected in 73.79 (*n* = 12), 12.64 (*n* = 11) and 8.05% (*n* = 7), respectively. As displayed in Table 2, a high variability in the combination of genes related with antibiotic resistance was observed, with *blaZ*, *tet*M, *nor*A, *vga*E, *dfr*A, and *dfr*K being the most detected pattern in 10.35% (*n* = 9) of the isolated MRSA.

### 3.3. Presence of Enterotoxin Genes

As shown in Table 2, enterotoxin genes were detected in 77.01% (*n* = 67). In spite of this high percentage, in most of the isolates just one gene was detected (34.48%, *n* = 30) and, overall, no more than four enterotoxin genes were detected in a single MRSA, isolates SA39 and SA67 being the only ones (Table 2). Specifically, the percentages of enterotoxin gene detection were as follows: *seb*_37.93% (*n* = 33), *see*_35.63% (*n* = 31), *sea*_31.03% (*n* = 27), *sec*_25.29% (*n* = 22) and *sed*_6.90% (*n* = 6).

Of note, all isolates with an absence of enterotoxin genes detected, namely, SA1, SA8, SA17, SA22, SA29, SA30, SA32, SA33, SA38, SA48, SA56, SA62, SA70 and SA87, were identified as multidrug resistant MRSA (Table 2, Table S2).

### 3.4. Molecular Subtyping of MRSA Isolates

MRSA isolates’ subtypes were ascertained by means of multilocus sequence typing (MLST) and *spa*-typing the isolates whose combination gave a total of 14 different MRSA genotypes (Table 3). On one hand, MLST outcomes showed that in most cases, positive isolates belonged to ST398 (82.76%, *n* = 72) followed by ST1 (9.20%, *n* = 8) and ST97 (4.60%, *n* = 4). Minor STs accounted for 1.15% of the positive samples (*n* = 1, each) belonging to ST30, ST4894 and ST5422 (Table 2 and Table 3).

On the other hand, a higher diversity of *spa* types was observed when compared with STs (Table 2 and Table 3). Specifically, 12 different types were detected, namely, the major *spa*-type recovered t899 (37.93%, *n* = 33) and t011 (31.03%, *n* = 27). The rest of *spa*-types were detected in a much lower proportion and classified as t127 (9.20%, *n* = 8), t18494 (4.60%, *n* = 4), t1730 (3.45%, *n* = 3), t4474 (2.30%, *n* = 2), t4795 (2.30%, *n* = 2), t1200 (2.30%, *n* = 2), t1939 (1.15%, *n* = 1), t1939 (1.15%, *n* = 1) and t4558 (1.15%, *n* = 1).

**Figure 2:**
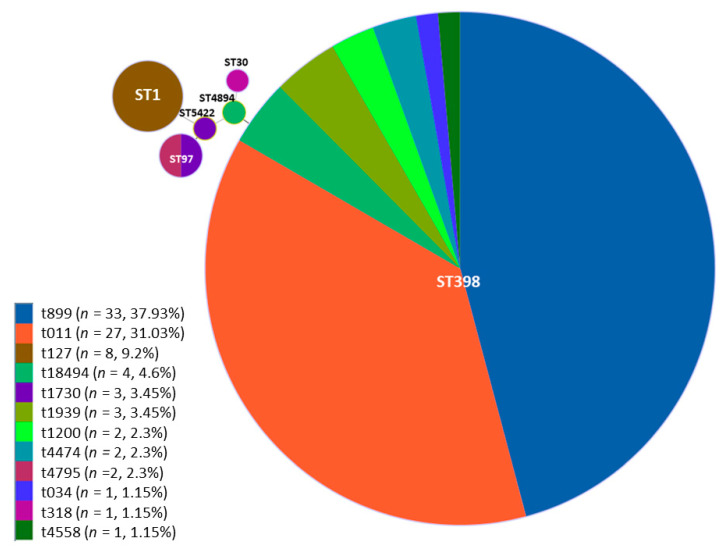
Minimum spanning tree of the 87 MRSA isolates analysed with MLST. The diagram was constructed by the full eBURST algorithm using the StaphType software v 2.0. Each single sequence type is depicted by a single circle and the size is proportional to the total number of isolates found within. Different colours in the circle indicate the different *spa* that are included in the same ST.

The full eBURST algorithm was used to describe the different STs based on their genetic proximity. As depicted in Figure 2, MRSA isolates were clustered into six different STs and, in particular, ST398 was the most variable, grouping eight different *spa*-types.

### 3.5. Biofilm Production Assays

In order to obtain a better picture of the phenotypic characteristics of the acquired isolates, biofilm production in polystyrene plates was determined through crystal violet staining [48]. Based on the outcomes obtained, 34.48% (*n* = 30) of the isolates were classified as non-biofilm producers, whereas 65.52% (*n* = 57) of the isolates were able to produce quantifiable amounts of biofilm at 24 h. Specifically, 44.83% (*n* = 39), were weak producers, 13.79% (*n* = 12), were moderate producers and 6.90% (*n* = 6), and were classified as strong producers (Table 2). The latter group included three environmental isolates (SA2, SA15 and SA18) and three animal isolates (SA7, SA8 and SA10) (Table 2).

Statistical analysis showed no correlation (*p* > 0.05) between the biofilm forming capacity (non-producer, weak, moderate and strong producer) and the antimicrobial multiresistance among the confirmed MRSA isolates.

## 4. Discussion

According to the latest report of The European Food Safety Authority (EFSA), MRSA prevalence in pig herds among European member states varied between 0.4 and 90.4% in 2017 with a slight reduction in 2018 with prevalence values ranging from 0 to 89.2% [49]. In the present work, 88 different swine farms at abattoir level were sampled obtaining a total of 440 samples. A total of 47.72% of the fattening units (*n* = 42) showed MRSA-positive results. It is important to note that this study was exclusively focused on heavy swine reared in Lombardy, the Italian region, with the main national pig production [50].

Similar to the outcomes obtained in the present work, results of positivity among pig farms in Italy have been also observed by Battisti et al. [22], in which a 38.1% of the sampled pig farms were positive for MRSA. Contrarily, recent studies performed in Italy showed a slightly higher incidence of MRSA-positive farms compared to the results obtained here. As a matter of illustration, the study of Parisi et al. [18] demonstrated that 64.7% of the pig farms turned out to be positive for MRSA. Similarly, Pirolo et al. [51] observed a positivity of MRSA in 66.8% of the swine farms sampled. These variations in results can be of a diverse nature, such as the use of different sampling, identification and characterisation methods that add an extra difficulty for prevalence data comparison [49,52].

Over the last years, the increasing resistance to antimicrobials among MRSA present in swine has been considered an issue of serious concern [21,53]. Antibiotic misuse/overuse in animal fattening units has posed an environmental pressure leading to the selection of a subpopulation of MRSA resistant not only to methicillin, but presenting cross-resistance to several other molecules [2,3,4,7]. This was observed by Alba et al. [54] in MRSA isolated from cattle farms in different European countries, demonstrating not only a high level of AMR, but the carriage of several virulence genes, conferring the fully invasive potential of MRSA to humans. In swine production chain, Lopes et al. [55] observed an unusual high level of resistance to clindamycin in the ST398 isolate, establishing a direct link between the use of antibiotics in a given farming unit and the level of resistance of the MRSA population present. Moreover, recently published data report a resistance to veterinary relevant antibiotics such as tiamulin of up to 50% among MRSA isolated from fattening pigs, and up to 100% among those isolated from pig meat [49]. This fact is in contrast with the obtained results, in which a slightly lower tiamulin level of resistance was observed (Figure 2).

As displayed by the results, a remarkable level of resistance to TET (Figure 2) and the presence of TET resistance-associated genes (Table 3), reported to be virtually present in all LA-MRSA [56,57], was demonstrated. This further corroborated data previously reported by several authors regarding TET resistance among LA-MRSA from pigs [18,22,23,51,57]. The main reason behind such TET-resistance levels is the intensive use of this antibiotic in farming [58,59,60] that may favour a cohort selection of TET-resistant bacteria; this is significantly notable in ST398 [21,53]. Taking a closer look, most of the obtained ST398 (CC398) isolates present TET resistance, which is in agreement with the latest EFSA data that claim that TET resistance is commonly associated with CC398 variants [49]. Nevertheless, among MRSA populations, a dynamic pattern is expected to be found due to natural evolution of this microorganism together with continuous exposure to antimicrobials and other environmental insults, thus modelling the overall phenotypic resistance characteristics of LA-MRSA in Italian abattoirs.

Multidrug resistance (MDR) was detected in 76.62% (*n* = 59) of the analysed MRSA (Table S2). The most observed resistance pattern was non-susceptibility to penicillin, amoxicillin/clavulanic acid, oxacillin, ampicillin, and ceftiofur. These observed high levels of resistance to penicillins and cephalosporins were in consonance with previously published data pointing out β-lactams resistance of MRSA in food systems [18,61]. It is important to remark that in this study two isolates, namely, SA63 (ST398/t899) and SA78 (ST1/t127), were found to display no susceptibility to 12 different molecules, respectively, representing 6 different classes. This is in line with previous results published in which the same MRSA genotypes isolated from the pork supply chain also displayed high levels of MDR [18]. This level of MDR encountered is of a special relevance when also considering the amount of resistance genes present among isolates and the fact that some of them are encoded in mobile elements that could further enhance horizontal and vertical resistance gene transfer in swine-related premises [56].

According to the EFSA report on antimicrobial monitoring on MRSA, the main route of transmission of LA-MRSA into the population is direct contact with animals, e.g., at abattoir level, and then dissemination [61]. In line with this, the study carried out by Pirolo et al. [62] involving pig farm workers demonstrated that 21.6% of the workers were colonised by livestock-associated MRSA (LA-MRSA) and that this colonisation was significantly higher if direct animal–human contact occurred. Additionally, Cuny et al. [63] detected a 30% antibiotic multiresistance to penicillins, tetracyclines and macrolides in ST398 MRSA isolated from humans in contact with farm animals, a fact that poses a therapeutic challenge if infection occurs. However, contamination routes within pig production environments still remain obscure considering that clones classified as community-acquired MRSA (CA-MRSA), such as ST1/t127 and ST30/t318, were also found among the MRSA isolated (Table 3), which is in agreement with previously reported data [64]. Regarding ST30, is important to remark that despite being commonly categorised as methicillin-sensitive *S. aureus* (MSSA) [65], MRSA variants have been isolated from pigs in Australia [66] and Portugal [67] that indicate not only a worldwide dissemination of this specific ST among pig herds, but tangible evidence of the dissemination of methicillin resistance via integration of the staphylococcal cassette chromosome *mec* (SCC*mec*) among sensitive strains [68]. Additionally, ST30 showed a remarkably high level of MDR (Table S2), harboured several antibiotic resistance and enterotoxin genes, and was identified as a strong biofilm producer (Table 2), which is highly relevant in the food production chain due to its potential harmfulness and environmental recalcitrance.

In European animal production systems, most efforts to track down MRSA routes of transmission have been mainly posed in regard to the swine production chain since it has been demonstrated that pigs are the major reservoir of several livestock-associated MRSA (LA-MRSA) genotypes such as ST97 [17,18,23], but especially ST398 [21,69]. Results obtained in this survey demonstrated that most of the MRSA isolates obtained belonged to the ST398, which is in agreement with the EFSA data and previous studies performed in Italy that highlight that this ST is the most prevalent LA-MRSA among pig herds [18,21,23,49]. Additionally, molecular characterisation detected eight different *spa*-types within ST398, including t011 and t899 with the latter being the most predominant, which is in line with previously reported data [18,22,53,69,70]. Of note, ST398/t899 have been reported to be among the major genotypes isolated in fattening pigs at abattoir level in Finland and Spain [49], indicative of a wide distribution regardless of the European zone. Additionally, in line with our results, recent data show that a high prevalence of ST398/t011 in Europe has been reported due to an increase in recent years among Swiss fattening pig herds [49]. Furthermore, a recent study conducted by Larsen et al. [71] demonstrated a high prevalence of ST398 in humans regardless of their direct contact with livestock animals, thus indicating that humans themselves could also act as further reservoirs for this specific lineage. It is important to highlight that ST398 was also present in 9 out of 10 of the environmental samples (Table 3).

ST97 was also found among the analysed samples (Table 2). In Italy, ST97 (t1730 and t4795) is considered to be the most prevalent swine-associated lineage after ST398 [22,72]. This LA-MRSA is commonly associated with infection in cattle, although spreading among pig herds in intensive farming have been also described [72]. It has been hypothesised that in pig farming, ST97 can acquire high levels of antibiotic resistance, as observed in our results (Table 1, Table S2), due to the selective pressure, and they can then be transmitted to humans, causing severe infections [72]. This capability of interspecies spreading together with the antimicrobial resistance makes ST97 an issue of serious concern for public health.

Genotype ST1/t127 was also detected (Figure 2, Table 3), which is in line with previously published data [21,22,53]. Although firstly categorised as community acquired (CA) MRSA [49,73] responsible for various infections in human [63,74,75], it has been also isolated from livestock animals [18,22,23]. With this regard, Franco et al. [76] carried out a phylogenetic characterisation of human and pig MRSA ST1/t127 isolates from Italy, Spain and Demark. The results obtained demonstrated that despite belonging to the same genotype, the homology between the two groups differed by 25%. Moreover, it was demonstrated that genotype ST1/t127 displayed a high resistance to major antimicrobials, which is in accordance with the results obtained in the present study (Table 3).

Staphylococcal enterotoxins (SEs) are considered one of the major virulence factors in *S. aureus* in the context of food safety [77]. This highly heat-resistant family of proteins can cause severe food poisoning outbreaks with symptoms including vomiting, abdominal cramping, and even fatality due to hypersensitivity induction [78], and their synthesis is dependent on the staphylococcal cellular density [79]. The latter aspect is considered within the EC Regulation 2073/2005 of microbiological safety of foodstuffs as a safety criterion in some food products of animal origin [80]. In this study, the enterotoxigenic potential of MRSA was evaluated, demonstrating that 77.01% of the isolates possessed at least one gene relate to SE synthesis (Table 3). This is in contrast with a previous study carried out by Normanno et al. [23], where no MRSA isolated from swine carried any SE gene. Similarly, Kluytmans [10] reported that most MRSA ST398 seldom carry SE genes, which clearly contrasts with the outcomes in this work since most of the isolates obtained carried at least one (Table 2). It is important to remark that among the isolated MRSA, a small proportion lacked on genes while presenting an MDR phenotype, and one of them, SA8, showed a high capacity of biofilm formation (Table 2). This aspect is important to take into account and highlights once more that the hazardous potential of MRSA strains found in swine production chain must be assessed from different angles, as a non-SE producer can harbour several resistance genes and become a persistent strain forming highly stable biofilms.

In scientific literature, it is widely accepted that *S. aureus* is able to embed itself in a polysaccharide matrix and form biofilms that adhere onto a wide variety of food-related surfaces, contributing to its survival to harsh environmental conditions (i.e., presence of antibiotics) alone or in multispecies communities [24,81]. The mechanisms beneath such resistance are diverse and lack antimicrobial diffusion into the biofilm matrix and the presence of recalcitrant cells, such as those belonging to the viable but not cultivable phenotype [82]. In *S. aureus*, the ability to form biofilms is directly related to the presence of virulence factors such as adhesins [83], and this structure contributes to the dissemination of resistance genes in the cells therein [5]. In this study, 65.52% of the resulting MRSA isolates were classified as biofilm-producing at 24 h. However, different levels of biofilm production were detected, in which most of the producers displayed low levels of biofilm production (i.e., weak producers, 44.83%) (see Section 3.4. for details). Similar results were previously reported by Di Ciccio et al. [84] in *S. aureus* strains from food and food-related environments. Of note, the quantification and subsequent classification of the biofilm capacity of the MRSA is only valid in the experimental conditions tested since, as demonstrated by Zhang et al. [83], biomass production, and subsequent classification, is directly dependent on the age of the biofilm. Based on our results, there was no correlation between the MDR phenotype and the capacity of a given MRSA of producing a biofilm. With this regard, a recent study carried out by Ou et al. [85] shows that there is a certain relationship between the level of antibiotic resistance and the biofilm forming ability in MRSA isolated from food from animals, but that the biofilm capacity of them is somehow impaired in those displaying the highest level of antibiotic resistance. Since MRSA biofilms can become recalcitrant contamination foci and a reservoir of several resistance genes, information about their adhesion capability is especially relevant to assess the potential hazard of a given subpopulation of MRSA, especially in those related with the food production chain.

## 5. Conclusions

Methicillin-resistant *Staphylococcus aureus* (MRSA) is among the major pathogens in the pig supply chain, and it is detected in both live and slaughtered animals as well as in meat products that are currently considered a potential MRSA transmission vector into the population [86,87]. In the present work, a high diversity of MRSA genotypes present in Italian pork supply chain was demonstrated. In addition to this, antimicrobial susceptibility evaluation highlighted high levels *β*-lactams and tetracyclines resistances among the isolated bacteria that may indicate an environmental selection of resistant MRSA variants due to the use of antibiotics in intensive animal farming. Additionally, we demonstrated capability of these MRSA to form biofilms, which gives these bacteria higher capability to survive in food-related premises. This could be translated into eventual recalcitrance and subsequent spreading of some genetic subtypes and antimicrobial resistance genes in the swine supply chain.

Lowering the level of antibiotic usage in order to improve animal and human welfare and thus avoid the selection process due to environmental pressure and dissemination of antibiotic resistances is an issue of extreme importance. Due to the high prevalence of some MRSA clones, such as ST398, it is of paramount importance to intensify specifically control measures regarding MRSA colonisation and transmission in farms and food-processing environments in order to avoid MRSA direct animal–human or foodborne spreading, through improvement of biosecurity practises, i.e., personal protection equipment, process environment and cleaning and disinfection procedures.

## Figures and Tables

**Table 1:** Major antimicrobial patterns and genotypes detected in multiresistant MRSA isolates.

Antibiotic Resistance Pattern	Genotypes (No. Isolates)
P-AMC-OX-AMP-TET-T	ST398/t011 (5); ST398/t899 (1); ST398/t4474 (1)
P-AMC-OX-AMP-FUR-TET-T	ST398/t011 (5)
P-AMC-OX-AMP-FUR-TET-ENR	ST398/t899 (8); ST97/t4795 (1)
P-AMC-OX-AMP-FUR-TET-DXT-T	ST398/t011 (6)
P-AMC-OX-AMP-FUR-KF-ENR-CN	ST1/t127 (3)
P-AMC-OX-AMP-FUR-TET-DXT-ENR	ST5422/t1730 (1); ST398/t1939 (1)
P-AMC-OX-AMP-KF-ENR-CN	ST1/t127 (2)
P-AMC-OX-AMP-FUR-TET-DXT-C-T	ST398/t011 (2)
P-AMC-OX-AMP-FUR-TET-C-FFC-CN-SxT	ST398/t899 (2)
P-AMC-OX-AMP-FUR-TET-DXT-C-FFC-SxT	ST398/t899 (2)
P-AMC-OX-AMP-FUR-TET-DXT-C-FFC-T-SxT	ST398/t899 (2)

P: penicillin; AMP: ampicillin; AMC: amoxicillin/clavulanic acid; OX: oxacillin; KF: cephalothin; FUR: ceftiofur; TET: tetracycline; DXT: doxycycline; ENR: enrofloxacin; C: chloramphenicol; FFC: florfenicol; CN: gentamycin; T: tiamulin; SxT: sulfamethoxazole/trimethoprim.

**Table 2:** Complete list of codes, sources, molecular characterisation and quantification of biofilm production of confirmed MRSA isolates obtained in this study.

Isolate ID	Source	ST	*spa*-Type	Antibiotic Resistance Genes	Enterotoxin Genes	Biofilm Production
SA1	Animal	398	t011	*blaZ*, *tet*L, *tet*M, *nor*A*, aad*D, *dfr*A, *dfr*K	n.d.	Moderate
SA2	Environment	398	t899	*blaZ*, *tet*L, *tet*M, *nor*A*, fex*A, *aac*A*aph*D, *aad*D, *vga*A, *dfr*A	*se* *b, sed, see*	Strong
SA3	Environment	398	t899	*blaZ*, *tet*L, *tet*M, *nor*A*, fex*A, *aad*D, *vga*A, *dfr*A	*se* *a, seb, see*	Weak
SA4	Environment	398	t899	*tet*L, *aad*D, *vga*A	*se* *b, see*	Weak
SA5	Animal	97	t4795	*blaZ*, *tet*L, *tet*M, *nor*A*, aad*D, *vga*A, *dfr*A	*se* *b, see*	Moderate
SA6	Animal	97	t4795	n.d.	n.d.	Moderate
SA7	Animal	5422	t1730	*blaZ*, *tet*M, *nor*A*, dfr*A,	*se* *a, seb, sed*	Strong
SA8	Animal	398	t899	*tet*M, *vga*A, *dfr*A, *dfr*K	n.d.	Strong
SA9	Animal	398	t011	*blaZ*, *tet*M, *vga*A, *dfr*A, *dfr*D	*seb*	Moderate
SA10	Animal	97	t1730	*blaZ*, *tet*L, *tet*M, *nor*A*, cfr*, *dfr*A, *dfr*D	*se* *a*	Strong
SA11	Animal	398	t899	*blaZ*, *tet*L, *tet*M, *fex*A, *aad*D, *vga*A, *dfr*A	*se* *a, seb*	Weak
SA12	Animal	398	t011	*blaZ*, *tet*L, *tet*M	*se* *a, seb*	Moderate
SA13	Environment	398	t1939	*blaZ*, *tet*M, *nor*A*, dfr*A	*se* *c*	Weak
SA14	Environment	398	t011	*tet*L	*se* *a, seb*	Non-producer
SA15	Environment	30	t318	*blaZ*, *tet*L, *tet*M, *fex*A, *cfr*, *aad*D, *dfr*A	*se* *a, seb*	Strong
SA16	Animal	398	t899	*blaZ*, *dfr*A	*se* *e*	Moderate
SA17	Environment	398	t1939	*blaZ*, *tet*M, *nor*A*, fex*A	n.d.	Non-producing
SA18	Environment	398	t034	*blaZ*, *tet*L, *tet*M, *nor*A*, fex*A, *cfr*, *aad*D, *dfr*A	*seb, see*	Strong
SA19	Environment	398	t18494	*blaZ*, *tet*M, *nor*A*, fex*A, *dfr*A	*se* *a, seb, see*	Weak
SA20	Environment	398	t1200	*tet*L*, fex*A, *cfr*, *aad*D, *dfr*K	n.d	Moderate
SA21	Animal	398	t1200	*blaZ*, *tet*M, *nor*A, *vga*E, *dfr*A, *dfr*K	*se* *b, sec*	Weak
SA22	Animal	398	t18494	*blaZ*, *tet*L, *tet*M, *vga*E, *dfr*A, *dfr*K	n.d.	Weak
SA23	Animal	398	t011	*tet*L, *tet*M, *aad*D, *vga*E, *dfr*A, *dfr*K	*se* *a, seb*	Weak
SA24	Animal	4894	t18494	*cfr*	*se* *a, sec, see*	Weak
SA25	Animal	398	t18494	*blaZ*, *tet*L, *tet*M, *cfr*, *dfr*D	*se* *a, seb, sec*	Weak
SA26	Animal	398	t899	*blaZ*, *tet*M, *nor*A*, vga*E, *dfr*A, *dfr*K	*se* *e*	Weak
SA27	Animal	398	t899	*blaZ*, *tet*M, *nor*A*, vga*E, *dfr*A, *dfr*K	*se* *c, see*	Weak
SA28	Animal	398	t899	*blaZ*, *tet*M, *nor*A*, vga*E, *dfr*A, *dfr*K	*se* *c*	Weak
SA29	Animal	398	t899	*blaZ*, *tet*M, *nor*A*, vga*E, *dfr*A, *dfr*K	n.d.	Moderate
SA30	Animal	398	t899	*blaZ*, *tet*M, *nor*A*, aad*D, *vga*E, *dfr*A, *dfr*K	n.d.	Weak
SA31	Animal	398	t899	*tet*M, *aad*D, *dfr*A	*se* *e*	Non-producer
SA32	Animal	97	t1730	*blaZ*, *tet*L, *tet*M, *nor*A, *aac*A*aph*D, *aad*D, *vga*A, *vga*E, *dfr*A, *dfr*K, *erm*T	n.d.	Weak
SA33	Animal	398	t011	*blaZ*, *tet*M, *nor*A*, vga*E, *dfr*A, *dfr*K	n.d.	Weak
SA34	Animal	398	t011	*blaZ*, *tet*M, *nor*A*, aad*D, *dfr*A, *dfr*K	*se* *c, see*	Weak
SA35	Animal	398	t899	*blaZ*, *tet*M, *nor*A, *vga*A, *vga*E, *dfr*A	*se* *c, see*	Weak
SA36	Animal	398	t899	*blaZ*, *tet*M, *nor*A*, aad*D, *vga*E, *dfr*A, *dfr*D, *dfr*K	*se* *a, seb, see*	Non-producer
SA37	Animal	398	t011	*blaZ*, *tet*M, *nor*A*, aad*D, *vga*E, *dfr*A, *dfr*D, *dfr*K	*se* *a, seb*	Weak
SA38	Animal	398	t011	*blaZ*, *tet*L, *tet*M, *nor*A, *aad*D, *dfrA*	n.d.	Non-producer
SA39	Animal	398	t011	*blaZ*, *tet*M, *nor*A*, aad*D, *vga*A, *vga*E, *dfr*A, *dfr*K	*se* *a, seb, sec, sed*	Weak
SA40	Animal	398	t011	*tet*L, *tet*M, *nor*A, *aad*D, *vga*E, *dfr*K	*se* *b*	Weak
SA41	Animal	1	t127	*blaZ*, *nor*A, *aac*A*aph*D, *aad*D, *vga*E, *dfr*A, *dfr*K, *erm*T	*se* *c*	Weak
SA42	Animal	1	t127	*blaZ*, *nor*A, *aac*A*aph*D, *aad*D, *vga*E, *dfr*A, *erm*T	*se* *b, sed*	Moderate
SA43	Animal	1	t127	*blaZ*, *nor*A, *aac*A*aph*D, *aad*D, *vga*E, *dfr*A, *erm*T	*se* *b, sec*	Weak
SA44	Animal	1	t127	*nor*A, *aac*A*aph*D, *vga*A, *vga*E, *dfr*A, *dfr*K, *erm*T	*se* *c*	Weak
SA45	Animal	1	t127	*nor*A, *aac*A*aph*D, *vga*A, *vga*E, *dfr*A	*se* *b, sec, see*	Non-producer
SA46	Animal	398	t899	*blaZ*, *tet*M, *nor*A*, vga*E, *dfr*A, *dfr*K	*se* *b*	Non-producer
SA47	Animal	398	t1939	*blaZ*, *tet*M, *fex*A, *cfr*, *aad*D, *vga*A, *vga*E, *dfr*A, *dfr*K	*se* *b, sec, see*	Non-producer
SA48	Animal	398	t899	*blaZ*, *fex*A, *aad*D, *vga*A, *vga*E, *dfr*A, *dfr*K	n.d.	Non-producer
SA49	Animal	398	t899	*blaZ*, *tet*L, *tet*M, *nor*A, *aac*A*aph*D, *aad*D, *vga*A, *vga*E, *dfr*A, *dfr*K	*se* *c, see*	Non-producer
SA50	Animal	398	t011	*blaZ*, *tet*M, *nor*A, *aad*D, *vga*E, *dfr*A	*se* *b, see*	Non-producer
SA51	Animal	398	t899	*blaZ*, *nor*A, *aad*D, *vga*E, *dfr*A, *dfr*D, *dfr*K	*se* *a, seb*	Moderate
SA52	Animal	398	t899	*tet*M, *nor*A, *aad*D, *vga*E, *dfr*A, *dfr*K	*se* *c, see*	Non-producer
SA53	Animal	398	t899	*blaZ, tet*M, *nor*A, *vga*A, *vga*E, *dfr*A, *dfr*K	*se* *b, sed, see*	Non-producer
SA54	Animal	398	t011	*blaZ, tet*M, *nor*A, *vga*A, *vga*E, *dfr*A, *dfr*K	*se* *e*	Non-producer
SA55	Animal	398	t011	*blaZ, tet*M, *nor*A, *vga*A, *vga*E	*se* *e*	Non-producer
SA56	Animal	398	t011	*blaZ, tet*M, *nor*A, *vga*A, *vga*E, *dfr*K	n.d.	Non-producer
SA57	Animal	398	t011	*blaZ*, *tet*M, *nor*A, *aad*D, *vga*E, *erm*T	*se* *b*	Non-producer
SA58	Animal	398	t4474	*blaZ*, *tet*M, *nor*A, *vga*A, *vga*E, *dfr*A	*se* *c, see*	Non-producer
SA59	Animal	398	t4474	*blaZ*, *tet*M, *nor*A, *vga*E, *dfr*A	n.d.	Weak
SA60	Animal	398	t011	*blaZ*, *tet*M, *nor*A, *vga*A, *vga*E, *dfr*A	*se* *e*	Non-producer
SA61	Animal	398	t011	*blaZ*, *tet*M, *nor*A, *vga*A, *dfr*A, *dfr*K	*se* *b, sec, see*	Weak
SA62	Animal	398	t899	*blaZ*, *tet*M, *nor*A, *vga*A, *vga*E, *dfr*A, *dfr*K	n.d.	Weak
SA63	Animal	398	t899	*blaZ*, *tet*L, *tet*M, *aac*A*aph*D, *aad*D, *vga*A, *vga*E, *dfr*A, *dfr*K, *erm*T	*se* *c*	Moderate
SA64	Animal	398	t899	*blaZ*, *tet*M, *nor*A, *dfr*A, *dfr*K	*se* *b, see*	Moderate
SA65	Animal	398	t899	*blaZ*, *tet*L, *tet*M, *aac*A*aph*D, *aad*D, *vga*A, *vga*E, *dfr*A, *dfr*K, *erm*T	n.d.	Weak
SA66	Animal	398	t4558	*blaZ*, *tet*M, *nor*A, *fex*A, *aac*A*aph*D, *vga*A, *vga*E, *dfr*A, *dfr*K	*se* *a, sec, sed, see*	Weak
SA67	Animal	398	t899	*blaZ*, *tet*M, *nor*A, *vga*E, *dfr*A, *dfr*K	*se* *c*	Weak
SA68	Animal	398	t899	*blaZ*, *tet*M, *nor*A, *vga*A, *vga*E, *dfr*A, *dfr*K	n.d.	Weak
SA69	Animal	398	t899	*blaZ*, *tet*M, *nor*A, *vga*A, *vga*E, *dfr*A, *dfr*K	*se* *c*	Weak
SA70	Animal	1	t127	*blaZ*, *nor*A, *aac*A*aph*D, *vga*A, *vga*E, *dfr*A, *dfr*K, *erm*T	n.d.	Weak
SA71	Animal	398	t011	*blaZ*, *tet*M, *nor*A, *aad*D, *vga*A, *vga*E, *dfr*A, *dfr*D	*se* *a*	Weak
SA72	Animal	398	t011	*tet*L, *tet*M, *nor*A, *aad*D, *vga*A, *vga*E, *dfr*A, *dfr*K	*se* *a*	Non-producer
SA73	Animal	398	t011	*tet*M, *nor*A, *aad*D, *vga*A, *vga*E, *dfr*D, *dfr*K	*se* *a*	Weak
SA74	Animal	398	t011	*tet*M, *nor*A*, aad*D, *vga*A, *vga*E, *dfr*A, *dfr*D, *dfr*K	*se* *b, see*	Non-producer
SA75	Animal	398	t011	*tet*L, *tet*M, *nor*A, *aad*D, *vga*A, *vga*E, *dfr*A, *dfr*K	*se* *a*	Weak
SA76	Animal	398	t011	*tet*M, *nor*A*, aad*D, *vga*E, *dfr*A, *dfr*D, *dfr*K	n.d.	Non-producer
SA77	Animal	1	t127	*blaZ*, *tet*L, *nor*A, *aac*A*aph*D, *aad*D, *vga*A, *vga*E, *dfr*A, *erm*T	*se* *a*	Weak
SA78	Animal	1	t127	*blaZ*, *tet*L, *nor*A, *aac*A*aph*D, *aad*D, *vga*A, *dfr*A, *dfr*K, *erm*T	*se* *a*	Weak
SA79	Animal	398	t899	*tet*M, *nor*A, *vga*E, *dfr*A	*se* *a*	Non-producer
SA80	Animal	398	t899	*bla*Z, *tet*M, *nor*A, *vga*E, *dfr*	*se* *a*	Non-producer
SA81	Animal	398	t899	*bla*Z, *tet*M, *nor*A, *vga*E, *dfr*	*se* *a, seb*	Non-producer
SA82	Animal	398	t899	*tet*M, *nor*A, *vga*E, *dfr*A	*se* *a, see*	Non-producer
SA83	Animal	398	t011	*blaZ*, *tet*M, *nor*A*, aad*D, *vga*E, *dfr*A, *dfr*D	*se* *e*	Non-producer
SA84	Animal	398	t899	*blaZ*, *tet*M, *nor*A*, vga*A, *vga*E	*se* *e*	Non-producer
SA85	Animal	398	t899	*blaZ*, *tet*M, *aad*D, *vga*A, *vga*E, *dfr*D	*se* *b*	Weak
SA86	Animal	398	t011	*blaZ*, *tet*M, *nor*A, *dfr*A	*se* *a*	Non-producer
SA87	Animal	398	t011	*blaZ*, *tet*M, *nor*A*, vga*E, *dfr*A	n.d.	Non-producer

n.d.: not detected.

**Table 3:** Molecular genotypes and frequencies observed among MRSA isolates in this study.

Genotype	Number of Isolates (%)
ST398/t899	33 (37.93%)
ST398/t011	27 (31.03%)
ST398/t1939	3 (3.45%)
ST398/t034	1 (1.15%)
ST398/t18494	3 (3.45%)
ST398/t1200	2 (2.30%)
ST398/t4474	2 (2.30%)
ST398/t4558	1 (1.15%)
ST1/t127	8 (9.20%)
ST97/t1730	2 (2.30%)
ST97/t4795	2 (2.30%)
ST4894/t18494	1 (1.15%)
ST30/t318	1 (1.15%)
ST5422/t1730	1 (1.15%)

## Supplementary Materials

The following is available online at https://www.mdpi.com/2304-8158/9/9/1141/s1, Table S1: List of primer sets used in this study; Table S2: Phenotypic antibiotic resistance pattern and minimum inhibitory concentrations (MIC) to human therapy and/or veterinary relevant antibiotics tested among isolated MRSA obtained in this study.
