# Supplementary material for: Assessment of the Antibiotic Resistance Profile, Genetic Heterogeneity and Biofilm Production of Methicillin-Resistant Staphylococcus aureus (MRSA) Isolated from The Italian Swine Production Chain
Source: Foods. 2020 Aug 19;9(9):1141. doi: 10.3390/foods9091141 (PMC7555242; doi:10.3390/foods9091141)
Supplement: Supplementary file 1 [file foods-09-01141-s001.zip › Table S1.docx]

**Table S1.** List of primer sets used in this study.

| **Target gene** | **Primer** | **Sequence 5'-3' sequence** | **Product (bp)** | **Reference** |
| --- | --- | --- | --- | --- |
| *aac*A*-aph*D | aacA-aphD-1  aacA-aphD-2 | CCA AGA GCA ATA AGG GCA TA  CAC TAT CAT AAC CAC TAC CG | 220 | [1] |
| *aad*D | aadD-1  aadD-2 | GCA AGG ACC GAC AAC ATT TC  TGG CAC AGA TGG TCA TAA CC | 165 | [1] |
| *bla*Z | blaZ-F  blaZ-R | CAG TTC ACA TGC CAA AGAG  TAC ACT CTT GGC GGT TTC | 762 | [1] |
| *cfr* | cfr-fw  cfr-rev | TGA AGT ATA AAG CAG GTT GGG AGT CA  ACC ATA TAA TTG ACC ACA AGC AGC | 746 | [2] |
| *dfr*A | dfrA-F  dfrA-R | CCT TGG CAC TTA CCA AAT G  CTG AAG ATT CGA CTT CCC | 374 | [1] |
| *dfr*D | dfrD-F  dFRd-R | TTC TTT AAT TGT TGC GAT GG  TTA ACG AAT TCT CTC ATA TAT ATG | 582 | [1] |
| *dfr*K | dfrK-1  dfrK-2 | GAG AAT CCC AGA GGA TTG GG  CAA GAA GCT TTT CGC TCA TAA A | 423 | [1] |
| *erm*T | ermT-1  ermT-2 | CCG CCA TTG AAA TAG ATC CT  TTC TGT AGC TGT GCT TTC AAA AA | 200 | [1] |
| *fex*A | fexA-fw  fexA-rv | GTA CTT GTA GGT GCA ATT ACG GCT GA  CGC ATC TGA GTA GGA CAT AGC GTC | 1272 | [3] |
| *mec*A | mecA1  mecA2 | GTA GAA ATG ACT GAA CGT CCG ATA A  CCA ATT CCA CAT TGT TTC CGT CTA A | 310 | [4] |
| *nuc* | nuc1  nuc2 | AGC CAA GCC TTG ACG AAC TAA ACG  GCG ATT GAT GGT GAT ACG GTT | 270 | [4] |
| *nor*A | nor2a  norA-5 | GTA ATA CCA GTC TTG CCT GT  GTA ATG GCT GGT CGT ATC AT | 878 | [5] |
| *se*A | GSEAr-1  GSEAR-2 | GGT TAT CAA TGT GCG GGT GG  CGG CAC TTT TTT CTC TTC GG | 102 | [6] |
| *se*B | GSEBR-1  GSEBR-2 | GTA TGG TGG TGT AAC TGA GC  CCA AAT AGT GAC GAG TTA GG | 164 | [6] |
| *se*C | GSECR-1  GSECR-2 | AGA TGA AGT AGT TGA TGT GTA TGG  CAC ACT TTT AGA ATC AAC CG | 451 | [6] |
| *se*D | GSEDR-1  GSEDR-2 | CCA ATA ATA GGA GAA AAT AAA AG  ATT GGT ATT TTT TTT CGT TC | 278 | [6] |
| *se*E | GSEER-1  GSEER-2 | AGG TTT TTT CAC AGG TCA TCC  CTT TTT TTT CTT CGG TCA ATC | 209 | [6] |
| *tet*L | tet(L)-1  tet(L)-2 | CAT TTG GTC TTA TTG GAT CG  ATT ACA CTT CCG ATT TCG G | 456 | [1] |
| *tet*M | tet(M)-1  tet(M)-2 | GTT AAA TAG TGT TCT TGG AG  CTA AGA TAT GGC TCT AAC AA | 576 | [1] |
| *vga*A | vga(A)inv-F  vga(A)inv-R | AGT GGT GGT GAA GTA ACA CG  CTT GTC TCC TCC GCG AAT AC | 659 | [7] |
| *vga*E | vga(E)-F  vga(E)-R | ATG AAA GAA TAG CAA TCC CAG  GGG TAG GTT GAG TTT GGA G | 541 | [8] |

**References**

1. Gómez-Sanz, E.; Torres, C.; Lozano, C.; Fernández-Pérez, R.; Aspiroz, C.; Ruiz-Larrea, F.; Zarazaga, M. Detection, molecular characterization, and clonal diversity of methicillin-resistant Staphylococcus aureus CC398 and CC97 in Spanish slaughter pigs of different age groups. *Foodborne Pathog. Dis.* **2010**, *7*, 1269–1277, doi:10.1089/fpd.2010.0610.

2. Inkster, T.; Coia, J.; Meunier, D.; Doumith, M.; Martin, K.; Pike, R.; Imrie, L.; Kane, H.; Hay, M.; Wiuff, C.; et al. First outbreak of colonization by linezolid- and glycopeptide-resistant Enterococcus faecium harbouring the cfr gene in a UK nephrology unit. *J. Hosp. Infect.* **2017**, *97*, 397–402, doi:10.1016/j.jhin.2017.07.003.

3. Kehrenberg, C.; Schwarz, S. Distribution of florfenicol resistance genes fexA and cfr among chloramphenicol-resistant Staphylococcus isolates. *Antimicrob. Agents Chemother.* **2006**, *50*, 1156–1163, doi:10.1128/AAC.50.4.1156-1163.2006.

4. McClure, J.A.; Conly, J.M.; Lau, V.; Elsayed, S.; Louie, T.; Hutchins, W.; Zhang, K. Novel multiplex PCR assay for detection of the staphylococcal virulence marker Panton-Valentine leukocidin genes and simultaneous discrimination of methicillin-susceptible from -resistant staphylococci. *J. Clin. Microbiol.* **2006**, *44*, 1141–1144, doi:10.1128/JCM.44.3.1141-1144.2006.

5. Vali, L.; Davies, S.E.; Lai, L.L.G.; Dave, J.; Amyes, S.G.B. Frequency of biocide resistance genes, antibiotic resistance and the effect of chlorhexidine exposure on clinical methicillin-resistant Staphylococcus aureus isolates. *J. Antimicrob. Chemother.* **2008**, *61*, 524–532, doi:10.1093/jac/dkm520.

6. Mehrotra, M.; Wang, G.; Johnson, W.M. Multiplex PCR for detection of genes for Staphylococcus aureus enterotoxins, exfoliative toxins, toxic shock syndrome toxin 1, and methicillin resistance. *J. Clin. Microbiol.* **2000**, *38*, 1032–1035, doi:10.1128/jcm.38.3.1032-1035.2000.

7. Lozano, C.; Aspiroz, C.; Rezusta, A.; Gómez-Sanz, E.; Simon, C.; Gómez, P.; Ortega, C.; Revillo, M.J.; Zarazaga, M.; Torres, C. Identification of novel vga(A)-carrying plasmids and a Tn5406-like transposon in meticillin-resistant Staphylococcus aureus and Staphylococcus epidermidis of human and animal origin. *Int. J. Antimicrob. Agents* **2012**, *40*, 306–312, doi:10.1016/j.ijantimicag.2012.06.009.

8. Argudín, M.A.; Vanderhaeghen, W.; Butaye, P. Diversity of antimicrobial resistance and virulence genes in methicillin-resistant non-Staphylococcus aureus staphylococci from veal calves. *Res. Vet. Sci.* **2015**, *99*, 10–16, doi:10.1016/j.rvsc.2015.01.004.
