# Supplementary material for: Assessment of the Antibiotic Resistance Profile, Genetic Heterogeneity and Biofilm Production of Methicillin-Resistant Staphylococcus aureus (MRSA) Isolated from The Italian Swine Production Chain
Source: Foods. 2020 Aug 19;9(9):1141. doi: 10.3390/foods9091141 (PMC7555242; doi:10.3390/foods9091141)
Supplement: Supplementary file 1 [file foods-09-01141-s001.zip › Table S2.docx]

**Table S2.** Phenotypic antibiotic resistance pattern and Minimum inhibitory concentrations (MIC) to human therapy and/or veterinary relevant antibiotics tested among isolated MRSA obtained in this study.

|  | Antibiotic susceptibility testing (Disk diffusion assay) | | | | | | | | | | | | | |  |  | MIC (mg/L) | | |  |  |
| --- | --- | --- | --- | --- | --- | --- | --- | --- | --- | --- | --- | --- | --- | --- | --- | --- | --- | --- | --- | --- | --- |
| Isolate ID | P30 | OX1 | AMC30 | AMP10 | KF30 | FUR30 | TE30 | DXT30 | ENR5 | C30 | FFC30 | CN10 | T30 | SxT25 | AMC | CN | DXT | ENR | FFC | SxT | TE |
| SA1 |  |  |  |  |  |  |  |  |  |  |  |  |  |  | 2 | 4 |  |  |  |  | > 256 |
| SA2 |  |  |  |  |  |  |  |  |  |  |  |  |  |  | 2 | 24 | 48 |  | 96 | > 32 | 192 |
| SA3 |  |  |  |  |  |  |  |  |  |  |  |  |  |  | 3 |  | 32 |  | 64 | >32 | > 256 |
| SA4 |  |  |  |  |  |  |  |  |  |  |  |  |  |  |  |  |  |  |  |  |  |
| SA5 |  |  |  |  |  |  |  |  |  |  |  |  |  |  | 2 |  |  | 3 |  |  | 96 |
| SA6 |  |  |  |  |  |  |  |  |  |  |  |  |  |  | 2 |  |  | 3 | 3 |  | > 256 |
| SA7 |  |  |  |  |  |  |  |  |  |  |  |  |  |  | 1 |  | 24 | 1,5 |  |  | 96 |
| SA8 |  |  |  |  |  |  |  |  |  |  |  |  |  |  | 8 |  | 12 |  | 96 | > 32 | > 256 |
| SA9 |  |  |  |  |  |  |  |  |  |  |  |  |  |  | 2 |  | 24 |  |  |  | > 256 |
| SA10 |  |  |  |  |  |  |  |  |  |  |  |  |  |  | 1,5 |  | 32 | 2 | 6 |  | 96 |
| SA11 |  |  |  |  |  |  |  |  |  |  |  |  |  |  | 6 |  | 48 |  | 96 | > 32 | > 256 |
| SA12 |  |  |  |  |  |  |  |  |  |  |  |  |  |  | 2 |  | 12 |  |  |  | 96 |
| SA13 |  |  |  |  |  |  |  |  |  |  |  |  |  |  | 1 |  | 8 | 12 |  |  | > 256 |
| SA14 |  |  |  |  |  |  |  |  |  |  |  |  |  |  |  |  |  |  |  |  |  |
| SA15 |  |  |  |  |  |  |  |  |  |  |  |  |  |  | 1,5 | 128 | 32 | >32 | > 256 |  |  |
| SA16 |  |  |  |  |  |  |  |  |  |  |  |  |  |  |  |  |  |  |  |  |  |
| SA17 |  |  |  |  |  |  |  |  |  |  |  |  |  |  |  |  |  |  |  |  |  |
| SA18 |  |  |  |  |  |  |  |  |  |  |  |  |  |  | 6 |  | 48 | > 32 | > 256 |  | 64 |
| SA19 |  |  |  |  |  |  |  |  |  |  |  |  |  |  | 1,5 | 192 | 16 |  | 96 |  | > 256 |
| SA20 |  |  |  |  |  |  |  |  |  |  |  |  |  |  |  |  |  |  | > 256 |  | 32 |
| SA21 |  |  |  |  |  |  |  |  |  |  |  |  |  |  |  |  |  |  |  |  |  |
| SA22 |  |  |  |  |  |  |  |  |  |  |  |  |  |  | 1,5 | 3 | 32 |  | 8 |  | > 256 |
| SA23 |  |  |  |  |  |  |  |  |  |  |  |  |  |  | 1,5 |  | 24 |  |  |  | 128 |
| SA24 |  |  |  |  |  |  |  |  |  |  |  |  |  |  |  |  |  |  |  |  |  |
| SA25 |  |  |  |  |  |  |  |  |  |  |  |  |  |  |  |  |  |  |  |  |  |
| SA26 |  |  |  |  |  |  |  |  |  |  |  |  |  |  | 0,5 |  |  | 2 |  |  | > 256 |
| SA27 |  |  |  |  |  |  |  |  |  |  |  |  |  |  | 0,5 |  |  | 4 |  |  | 64 |
| SA28 |  |  |  |  |  |  |  |  |  |  |  |  |  |  | 0,5 |  |  | 6 |  |  | > 256 |
| SA29 |  |  |  |  |  |  |  |  |  |  |  |  |  |  | 0,38 |  |  | 3 |  |  | > 256 |
| SA30 |  |  |  |  |  |  |  |  |  |  |  |  |  |  | 0,75 |  |  | 4 |  |  | 128 |
|  | Antibiotic susceptibility testing (Disk diffusion assay) | | | | | | | | | | | | | | MIC (mg/L) | | | | | | |
| Isolate ID | P30 | OX1 | AMC30 | AMP10 | KF30 | FUR30 | TE30 | DXT30 | ENR5 | C30 | FFC30 | CN10 | T30 | SxT25 | AMC | CN | DXT | ENR | FFC | SxT | TE |
| SA31 |  |  |  |  |  |  |  |  |  |  |  |  |  |  |  |  |  |  |  |  |  |
| SA32 |  |  |  |  |  |  |  |  |  |  |  |  |  |  | 2 | 16 |  |  |  |  | 64 |
| SA33 |  |  |  |  |  |  |  |  |  |  |  |  |  |  | 2 |  |  |  |  |  | 64 |
| SA34 |  |  |  |  |  |  |  |  |  |  |  |  |  |  | 1,5 |  |  |  |  |  | 128 |
| SA35 |  |  |  |  |  |  |  |  |  |  |  |  |  |  |  |  |  |  |  |  |  |
| SA36 |  |  |  |  |  |  |  |  |  |  |  |  |  |  |  |  |  |  |  |  |  |
| SA37 |  |  |  |  |  |  |  |  |  |  |  |  |  |  | 4 |  | 32 |  |  |  | 96 |
| SA38 |  |  |  |  |  |  |  |  |  |  |  |  |  |  | 3 |  | 24 |  |  |  | 96 |
| SA39 |  |  |  |  |  |  |  |  |  |  |  |  |  |  | 1 | 96 |  |  |  |  | > 256 |
| SA40 |  |  |  |  |  |  |  |  |  |  |  |  |  |  | 2 |  | 32 |  |  |  | > 256 |
| SA41 |  |  |  |  |  |  |  |  |  |  |  |  |  |  |  |  |  |  |  |  |  |
| SA42 |  |  |  |  |  |  |  |  |  |  |  |  |  |  | 32 | 5 |  | 4 |  |  |  |
| SA43 |  |  |  |  |  |  |  |  |  |  |  |  |  |  | 16 | 12 |  | 3 |  |  |  |
| SA44 |  |  |  |  |  |  |  |  |  |  |  |  |  |  | 48 | 24 |  | 6 |  |  |  |
| SA45 |  |  |  |  |  |  |  |  |  |  |  |  |  |  | 24 | 24 |  | 4 |  |  |  |
| SA46 |  |  |  |  |  |  |  |  |  |  |  |  |  |  | 0,19 |  |  | 0,25 |  |  | 256 |
| SA47 |  |  |  |  |  |  |  |  |  |  |  |  |  |  | 0,19 |  |  |  | 48 |  | 64 |
| SA48 |  |  |  |  |  |  |  |  |  |  |  |  |  |  | 4 | 2 |  |  | 96 | 24 | 64 |
| SA49 |  |  |  |  |  |  |  |  |  |  |  |  |  |  | 3 |  |  |  |  |  | > 256 |
| SA50 |  |  |  |  |  |  |  |  |  |  |  |  |  |  | 1,5 |  |  |  |  |  | 32 |
| SA51 |  |  |  |  |  |  |  |  |  |  |  |  |  |  | 3 |  | 48 |  | 96 | > 32 | 96 |
| SA52 |  |  |  |  |  |  |  |  |  |  |  |  |  |  | 0,75 |  |  | 3 |  |  | 96 |
| SA53 |  |  |  |  |  |  |  |  |  |  |  |  |  |  | 1,5 |  |  |  |  |  | 128 |
| SA54 |  |  |  |  |  |  |  |  |  |  |  |  |  |  | 3 |  |  |  |  |  | 192 |
| SA55 |  |  |  |  |  |  |  |  |  |  |  |  |  |  | 4 |  |  |  |  |  | 96 |
| SA56 |  |  |  |  |  |  |  |  |  |  |  |  |  |  | 4 |  | 32 |  |  |  | 192 |
| SA57 |  |  |  |  |  |  |  |  |  |  |  |  |  |  |  |  |  |  |  |  |  |
| SA58 |  |  |  |  |  |  |  |  |  |  |  |  |  |  |  |  |  |  |  |  |  |
| SA59 |  |  |  |  |  |  |  |  |  |  |  |  |  |  | 0,75 |  |  |  |  |  | > 256 |
| SA60 |  |  |  |  |  |  |  |  |  |  |  |  |  |  | 1,5 |  |  |  |  |  | 96 |
| SA61 |  |  |  |  |  |  |  |  |  |  |  |  |  |  | 3 |  |  |  |  |  | > 256 |
| SA62 |  |  |  |  |  |  |  |  |  |  |  |  |  |  |  | 2 |  | 0,38 |  |  | 96 |
|  | Antibiotic susceptibility testing (Disk diffusion assay) | | | | | | | | | | | | | | MIC (mg/L) | | | | | | |
| Isolate ID | P30 | OX1 | AMC30 | AMP10 | KF30 | FUR30 | TE30 | DXT30 | ENR5 | C30 | FFC30 | CN10 | T30 | SxT25 | AMC | CN | DXT | ENR | FFC | SxT | TE |
| SA63 |  |  |  |  |  |  |  |  |  |  |  |  |  |  | 2 | 4 |  | 0,38 | 48 | > 32 | 64 |
| SA64 |  |  |  |  |  |  |  |  |  |  |  |  |  |  |  |  |  |  |  |  |  |
| SA65 |  |  |  |  |  |  |  |  |  |  |  |  |  |  | 2 | 32 |  |  | 96 | > 32 | 96 |
| SA66 |  |  |  |  |  |  |  |  |  |  |  |  |  |  | 6 | 24 |  | 12 | 96 |  | 24 |
| SA67 |  |  |  |  |  |  |  |  |  |  |  |  |  |  |  |  |  |  |  |  |  |
| SA68 |  |  |  |  |  |  |  |  |  |  |  |  |  |  |  | 2 | 12 |  |  |  | 192 |
| SA69 |  |  |  |  |  |  |  |  |  |  |  |  |  |  |  | 2 |  |  |  |  | 96 |
| SA70 |  |  |  |  |  |  |  |  |  |  |  |  |  |  | 24 | 32 |  | 8 |  |  |  |
| SA71 |  |  |  |  |  |  |  |  |  |  |  |  |  |  | 4 |  |  |  |  |  | > 256 |
| SA72 |  |  |  |  |  |  |  |  |  |  |  |  |  |  | 6 |  | 24 |  |  |  | 128 |
| SA73 |  |  |  |  |  |  |  |  |  |  |  |  |  |  | 1,5 | 1,5 | 16 |  |  |  | 128 |
| SA74 |  |  |  |  |  |  |  |  |  |  |  |  |  |  |  |  |  |  |  |  |  |
| SA75 |  |  |  |  |  |  |  |  |  |  |  |  |  |  | 3 | 6 |  |  |  |  | 128 |
| SA76 |  |  |  |  |  |  |  |  |  |  |  |  |  |  | 1 |  |  |  |  |  | > 256 |
| SA77 |  |  |  |  |  |  |  |  |  |  |  |  |  |  |  |  |  |  |  |  |  |
| SA78 |  |  |  |  |  |  |  |  |  |  |  |  |  |  | 24 | 2 |  | 2 | > 256 |  | 6 |
| SA79 |  |  |  |  |  |  |  |  |  |  |  |  |  |  |  |  |  |  |  |  |  |
| SA80 |  |  |  |  |  |  |  |  |  |  |  |  |  |  |  |  |  |  |  |  |  |
| SA81 |  |  |  |  |  |  |  |  |  |  |  |  |  |  | 1 |  |  |  |  | 4 | 64 |
| SA82 |  |  |  |  |  |  |  |  |  |  |  |  |  |  |  |  |  |  |  |  |  |
| SA83 |  |  |  |  |  |  |  |  |  |  |  |  |  |  | 3 |  |  |  |  |  | 192 |
| SA84 |  |  |  |  |  |  |  |  |  |  |  |  |  |  | 1 |  |  | 4 |  |  | 96 |
| SA85 |  |  |  |  |  |  |  |  |  |  |  |  |  |  |  |  |  |  |  |  |  |
| SA86 |  |  |  |  |  |  |  |  |  |  |  |  |  |  | 1,5 |  | 12 |  |  |  | > 256 |
| SA87 |  |  |  |  |  |  |  |  |  |  |  |  |  |  | 2 |  | 16 |  |  |  | 128 |
| Total | 80 | 83 | 80 | 82 | 11 | 42 | 74 | 28 | 24 | 21 | 19 | 22 | 32 | 9 |  |  |  |  |  |  |  |
| % | 91,95 | 95,40 | 91,95 | 94,25 | 12,64 | 48,28 | 85,06 | 32,18 | 27,59 | 24,14 | 21,84 | 25,29 | 36,78 | 10,34 |  |  |  |  |  |  |  |

Antibiotics tested: P: penicillin; AMP: ampicillin; AMC: amoxicillin-clavulanic acid; OX: oxacillin; KF: Cephalotin; FUR: ceftiofur; TET: tetracyclin; DXT: doxycyclin; ENR: enrofloxacin; C: chloramphenicol; FFC: florfenicol; CN: Gentamycin; T: Tiamulin; SxT: Sulfamethoxazole-Trimethoprim. Grey squares mean resistance to a given antibiotic determined in the disk diffusion assay. Numbers next to the antibiotics’ acronyms, make reference to the disk load in µg. Of note, MIC assays were only perfomed in those isolates considered as multiresistant (i.e. displaying resistance to 3 or more antibiotic classes).
